# Low dose ATG-Fresenius for GVHD prophylaxis: a comparative study with ATG-Thymoglobulin

**DOI:** 10.3389/fimmu.2025.1526513

**Published:** 2025-01-27

**Authors:** Itai Falicovich, Boaz Nachmias, Shlomo Elias, Eran Zimran, Adir Shaulov, Polina Stepensky, Batia Avni, Sigal Grisariu

**Affiliations:** ^1^ Faculty of Medicine, The Hebrew University, Jerusalem, Israel; ^2^ Department of Hematology, Hadassah Medical Center and Faculty of Medicine, Hebrew University, Jerusalem, Israel; ^3^ Bone Marrow Transplantation and Cancer Immunotherapy Department, Hadassah University Medical Center and Faculty of Medicine, The Hebrew University, Jerusalem, Israel

**Keywords:** ATG Fresenius, ATG thymoglobulin, allogenic bone marrow transplantation, acute GVHD, chronic GVHD

## Abstract

**Background:**

Anti-Thymocyte Globulin (ATG) is commonly used to prevent graft-versus-host disease (GVHD), but the optimal dosage and type of ATG remains to be determined.

**Objective:**

We compared retrospectively the safety and efficacy outcomes of allogeneic transplantation using low-dose ATG-Fresenius (15mg/kg) and ATG-Thymoglobulin (10mg/kg) for GVHD prevention.

**Study design:**

Ninety-eight patients were included, with 46 in the ATG-T group and 52 in the ATG-F group. The median age was 48 years in the ATG-T group (range 20-71) and 50 years in the ATG-F group (range 18-73). Baseline characteristics were similar, with slightly more HLA mismatched donors and single-agent cyclosporine GVHD prophylaxis use in the ATG-T group. Additionally, the ATG-F group had more myeloid leukemia and myelodysplastic syndrome patients, while the ATG-T group had more lymphoma patients.

**Results:**

The cumulative incidence of acute GVHD (aGVHD) grade II-IV and chronic GVHD (cGVHD) showed no significant differences. Multivariate analysis indicated that donor HLA mismatch influenced aGVHD risk significantly (p=0.005), and myeloablative conditioning increased cGVHD risk. Bacteremia and CMV reactivation rates were similar, but EBV DNA viremia was higher in the ATG-T group (22% *vs*. 8%, p=0.047), with one case of Post-Transplant Lymphoproliferative Disorder (PTLD) in the ATG-T group. Cumulative incidence of overall survival (OS), relapse incidence, non-relapse mortality (NRM) and GVHD free, Relapse free Survival (GRFS) did not significantly differ.

**Conclusions:**

This study highlights the safety and efficacy of low-dose ATG-F compared to a relatively high dose ATG-T. Prospective studies are necessary to validate the safety and efficacy of low dose ATG-F for GVHD prevention.

## Introduction

Anti-Thymocyte Globulin (ATG) is frequently employed in the prevention of Graft Versus Host Disease (GVHD) as well as graft failure. It functions as an immunoregulator by attaching to T-cells and various other immune system cells (1). Among available ATG sera are ATG-Thymoglubolin (ATG-T, Sanofi Genzyme, Cambridge MA), derived from rabbit vaccination with human thymocytes, and ATG-Fresenius (ATG-F, Neovii, Rapperswil, Switzerland, ATG Fresenius^®^), derived from the human Jurkat T-cell line. While numerous clinical studies have demonstrated the effectiveness of each of these agents individually (2–8), there is a paucity of studies directly comparing the two agents. Furthermore, the variability in dosing regimens adds complexity to the comparison between these treatments.

The optimal dosage of ATG for GVHD prophylaxis displays variability, as demonstrated in multiple studies. An in-depth analysis of ATG formulations has unveiled distinctions in the targeted antigens between ATG-Fresenius and Thymoglobulin, potentially contributing to variations in their immunomodulatory capabilities (9). Since ATG-F recognizes a more limited spectrum of antigens, higher doses are used to achieve adequate immunomodulation compared to ATG-T. Additionally, it has been observed that different ATG products exhibit differing clearance rates, further influencing their immunomodulatory effects (10). Furthermore, the significance of patient-specific factors, such as absolute lymphocyte counts (ALCs), has been underscored, with individuals possessing lower ALCs being susceptible to receiving excessive ATG doses, resulting in profound T-cell depletion and inferior outcomes (11). These findings shed light on just a subset of the factors contributing to differences in these two formulations and their respective dosing regimens.

Reported ATG-T doses ranges from 2.5 to 10mg/kg (12). High doses of ATG-T (15mg/kg) compared to no ATG have been shown to reduce the incidence of acute GVHD (aGVHD) (50% *vs*. 11%, p=0.001) and chronic GVHD (cGVHD) (62% *vs* 39%; P =.04), while exposing the patients to a higher incidence of lethal infections (30% *vs* 7%, p=0.02) [3]. Lower doses of 4.5 mg/kg (again compared to no ATG) in patients who underwent hematopoietic stem cell transplantation (HSCT) from an HLA matched unrelated donor (MUD) was associated with a reduction of acute and chronic GVHD incidence, reduced use of post-transplant immunosuppression therapy (IST), and reduced patients’ symptoms burden, but with an increased incidence of EBV infections (5). Similar effects have been observed in larger prospective studies of HSCT from HLA matched sibling donors (MSD) (13) and MUD (6), demonstrating a reduced incidence of aGVHD and cGVHD, without significant differences in incidence of infections compared to control groups.

Similarly, ATG-F doses range widely between 15mg/kg to 60mg/kg (14). A phase 3 randomized study demonstrated that lower doses of ATG-F (15mg/kg compared to 30mg/kg) reduced relapse incidence and increased five-year overall survival (OS) in pediatric patients undergoing allogeneic HSCT from MUD with a myeloablative conditioning regimen (15). In adults, the optimal dose of ATG-F has not yet been defined. A phase 3 randomized controlled study assessed the efficacy and safety of prophylactic ATG-F (at a total dose of 60 mg/kg) in adult patients undergoing allogeneic HSCT compared with no ATG (4). In the group of patients receiving ATG-F there was a significant reduction in the incidence of grade II-IV aGVHD and extensive cGVHD, without an increase in relapse or non-relapse mortality. Others have reported a lower rate of cGVHD with low dose (15-30 mg/kg) of ATG-F (16, 17).

Recently, two retrospective studies compared transplant outcomes between the two agents. Both studies showed a statistically significant decline in the incidence of overall cGVHD and moderate-severe cGVHD in patients who received ATG-F (at a dosage of 30mg/kg and 20 mg/kg, respectively) compared to those who received ATG-T (7.5mg/kg and 10mg/kg, respectively). There was no significant difference in the rate of aGVHD or infectious complications (18, 19).

In our clinical practice, between the years 2011-2014, we administered ATG-T at a dosage of 10mg/kg. As safety data for ATG-F accumulated, suggesting lower incidence of infectious complication due to the narrower antigen spectrum, we switched to ATG-F at a dosage of 15mg/kg starting in 2014 onwards. Despite previous studies showing the efficacy of ATG-F (60mg/kg) (4), we have chosen a lower dose of ATG-F to mitigate concerns regarding an increased risk of infection and relapse (3, 5, 15). We conducted a retrospective study at our center comparing transplant outcomes using ATG-F 15mg/kg (from 2014 forward) to our earlier protocol using ATG-T 10mg/kg. Given the lack of outcome data comparing between these two agents at these dosages, this study aims to address the gap and provide valuable insights into their relative efficacy and toxicity.

## Methods

The study cohort included all patients above the age of 18 years old who underwent HSCT with ATG-T or ATG-F as GVHD prophylaxis at Hadassah university medical center from 2011-2018. Patients with an underlying disease for which the choice of ATG type has remained ATG-T (i.e., aplastic anemia), were not included in the study population. Data collected included patients’ demographics, diagnosis, treatment outcomes and infectious complications. Adverse events were graded according to the CTCAE 4.0. The follow-up period spanned two years.

Myeloablative regimens included: Total body irradiation (TBI) ≥ 500 cGy as a single fraction or ≥ 800cGy if fractionated, total busulfan ≥ 9mg/kg, total melphalan ≥ 150mg/m^2^, total Thiotepa ≥ 10mg/kg and treosulfan ≥36g/m2/d. Any other conditioning regimen utilized was categorized under the reduced-intensity regimen. ATG was administered to patients transplanted for MDS regardless of donor type and those transplanted from unrelated donors (both HLA matched and HLA mismatched). ATG-T was administered at a dosage of 2.5mg/kg/d for four consecutive days (on days -4, -3, -2, -1). ATG-F was administered at a dosage of 5mg/kg/d for three consecutive days (on days -3, -2, -1). The initial target for cyclosporine trough levels was 200-300ng/ml during the first month, and it was subsequently lowered to a range of 100-150ng/ml thereafter. Mycophenolate Mofetil (MMF) was initially given at a dose of 15mg/kg three times daily during the first month and then gradually tapered down. Neutrophil engraftment was defined as the first of three consecutive days with an absolute neutrophil count > 0.5 per microliter (mcL). Platelet engraftment was defined as the first of seven consecutive days with a platelet count > 20 per mcL, without platelet transfusion. Post-transplant donor chimerism was monitored using peripheral blood (PB) and bone marrow (BM) short tandem repeats (STR) analysis. Acute and chronic GVHD were graded according to Mount Sinai Acute GvHD International Consortium (MAGIC) criteria for acute GVHD (20) and the NIH 2014 criteria for chronic GvHD previously published criteria (21). cGVHD incidence was calculated for patients surviving more than 100 days. Overall survival (OS) was defined as time from transplant to death from any cause. Non- Relapse mortality (NRM) was defined as mortality without prior relapse. GVHD and relapse-free survival (GRFS) was evaluated as a composite end point of: absence of grades III–IV acute GVHD, moderate-severe chronic GVHD requiring systemic immunosuppressive therapy, relapse, or death from any cause, during any time point after allo-HSCT.

The study was approved by the Hadassah University Hospital review board and was performed in accordance with the principles of the Declaration of Helsinki. Informed consent was waived by the Hadassah University Hospital ethical committee (approval no. 0608-20-HMO).

### Statistical analysis

To test the association between two categorical variables, the χ^2^ test as well as the Fisher’s exact test was used. The comparison of a quantitative variable between two independent groups was performed by using the two-sample t-test or the non-parametric Mann-Whitney test for variables which were not normally distributed. The Kaplan-Meier survival model was used for testing the effect of categorical variables on survival, with the log-rank test for the comparison of survival curves. The Cox regression model was applied for testing the effect of quantitative variables on survival. This model was also used as the multivariate model for survival. The multivariable model included 2 blocks. In the first block, ATG type was forced into the regression and in the second block using the stepwise, forward, likelihood ratio approach, only significant pre transplant risk factors (such as demographic, underlying disease and pre-transplant characteristics, including median recipient age, gender, disease status at transplantation entry and comorbidity index, donor type, donor age and gender matching, HLA matching, ABO and CMV serology matching, transplant source, conditioning regimen intensity and GVHD prophylaxis) identified in the univariate analysis were incorporated in the Cox regression model. Probabilities of NRM, relapse and GVHD were calculated using the cumulative incidence function, accounting for competing risks, and were compared using Gray’s test. Relapse was the competing risk of NRM and vice versa, and death was the competing risk of GVHD. All statistical tests used were two-tailed, and a p-value of 0.05 or less was considered statistically significant. The statistical analysis was performed using SPSS 26 and NCSS 24 software.

## Results

### Patient characteristics

Ninety-eight patients were included in the study, 46 in the ATG-T group and 52 in the ATG-F group. Baseline clinical characteristics of the groups are summarized in **Table 1**. Median follow up was 7.26 months in the ATG-T group *vs*. 14.58 months in the ATG-F group (p=0.276). Demographic, underlying disease and pre-transplant characteristics, including median recipient age, gender, disease status at transplantation entry, calculated refined disease risk index (rDRI) (22) and comorbidity index of the two groups were mostly similar. There was a significant difference between the two groups regarding underlying disease leading to transplantation (p=0.038, Chi-Square test), stemming from a higher percentage of patients transplanted for MDS and secondary AML in the ATG-F compared to the ATG-T group (21% Vs. 11%, respectively) and a higher rate of lymphoproliferative diseases (other than acute lymphoblastic leukemia) in the ATG-T compared to the ATG-F group (15% Vs. none, respectively).

**Table 1:** Patient characteristics.

Variables		ATG-T (n=46)	ATG-F (n=52)	p-value
**Gender**	Male	30 (65.2%)	39 (75%)	0.29
	Female	16 (34.8%)	13 (25%)	
**Age at transplant (median), years**		48.09 (19.9-70.9)	50.51 (18.38-72.9)	0.258
**Donor age (median), years**		30 (16-77)	27 (15-66)	0.441
**Underling Disease**	AML	17 (37%)	18 (34.6%)	**0.038**
	SecAML	8 (17.4%)	14 (36.9%)	
	ALL	5 (10.9%)	7 (13.5%)	
	MDS	5 (10.9%)	11 (21.2%)	
	MPN	1 (2.2%)	1 (1.9%)	
	LPD	7 (15.2%)	0	
	Others	3 (6.5%)	1 (1.9%)	
**Disease status at Tx**	CR	21 (45.7)	30 (57.7%)	0.497
	PR	2 (4.3%)	2 (3.8%)	
	AD	23 (50%)	20 (38.5)	
**HCT-CI**	Low (0)	8 (17.4%)	8 (15.4%)	0.627
	Moderate (1-2)	24 (52.2%)	32 (65.1%)	
	High (≥3)	14 (30.4%)	12 (23.1%)	
**rDRI**	Low-Intermediate	28 (60.9%)	35 (67.3%)	0.533
	High-Very High	18 (39.1%)	17 (32.7%)	
**Donor Type**	Sibling	9 (19.6%)	13 (25%)	0.630
	Unrelated	37 (80.4%)	38 (73.1%)	
	Other related	0	1 (1.9%)	
**Transplant source**	PBSC	41 (89.1%)	49 (94.2%)	0.469
	BM	5 (10.9%)	3 (5.8%)	
**HLA matching**	Match	28 (60.9%)	41 (78.8%)	**0.052**
	Mismatch	18 (39.1%)	11 (21.2%)	
**ABO incompatibility**	Matched	16 (34.8%)	25 (49.0%)	0.438
	Minor	13 (28.3%)	14 (27.5%)	
	Major	12 (26.1%)	9 (17.6%)	
	Bidirectional	5 (10.9%)	3 (5.9%)	
**Conditioning regimen**				
	MA	29 (63%)	26 (50%)	
	RIC	17 (37%)	26 (50%)	
**GVHD prophylaxis**	CSA	14 (30.4%)	2 (3.8%)	**<0.01**
	CSA+MTX	0	2 (3.8%)	
	CSA+MMF	32 (69.6%)	48 (92.3%)	
**Gender matching D/R**	M/F	10 (21.7%)	8 (15.4%)	0.695
	F/F	6 (13.0%)	5 (9.6%)	
	M/M	18 (39.1%)	26 (50.0%)	
	F/M	12 (26.1%)	13 (25.0%)	
**CMV – D/R serology status**	+/+	28 (60.9%)	42 (82.4%)	**0.022**
	+/-	1 (2.2%)	2 (3.9%)	
	-/+	14 (30.4%)	7 (13.7%)	
	-/-	3 (6.5%)	0	

AML, Acute Myeloid Leukemia; secAML, Secondary AML; ALL, Acute Lymphoid Leukemia; MDS, Myelodysplastic syndrome; MPN, myeloprolipherative Disorder; LPD, lymphroliferative Disorder; Tx, Treatment; CR, Complete Remission; PR, Partial Remission; AD, Active Disease; HCT, CI Hematopoietic Cell Transplantation Comorbidity Index; rDRI, Refined Disease Risk Index; PBSC, Peripheral Blood Stem Cell; BM, Bone Marrow; MA, myeloablative; RIC, reduced intensity; CSA, Cyclosporin A; MMF, Mycophenolate Mofetil; D/R, Donor/Recipient.Bold p-values signify statistical significance.

Addressing known risk factors for GVHD (**Table 1**), there was no significant difference between the two cohorts regarding the median donor age and conditioning regimen intensity. However, there was a borderline significant higher incidence of HLA mismatch in the ATG-T group compared to the ATG-F group (39.1% Vs. 21.2%; p=0.052, Chi-Square test). In addition, a significantly higher number of patients received single-agent cyclosporine in the ATG-T compared to the ATG-F group (30.4% *vs* 3.8%, respectively; p<0.01, Chi-Square test). This difference is primarily attributed to the use of ATG-T during an earlier (before 2014) timeframe.

Regarding risk factors for infections, there was a significantly higher incidence of positive IgG serology for CMV in both donors and recipients within the ATG-F compared to the ATG-T cohort (82.4% *vs* 60.9%, respectively, p=0.022, Fisher-Freeman-Halton Exact test).

### GVHD

**Figure 1:**
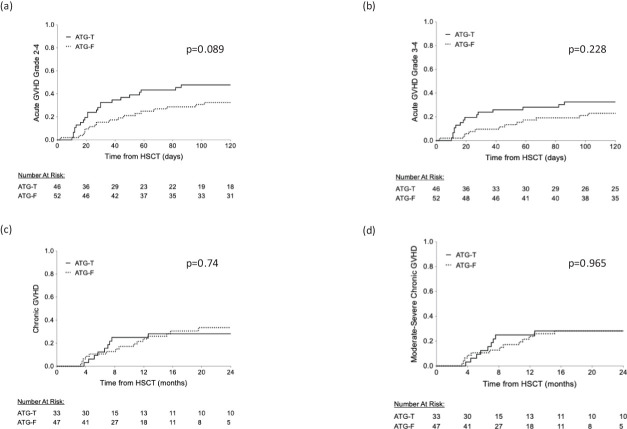
Cumulative rate of acute and chronic GVHD. **(A)** Acute GVHD grade II-IV **(B)** Acute GVHD grade III-IV **(C)** chronic GVHD **(D)** moderate-severe chronic GVHD.

aGVHD grade II-IV occurred in 23 out of 46 patients in the ATG-T group versus 21 out of 52 patients in the ATG-F group (50% *vs* 40.4%, p=0.417, Chi-Square test). The proportions of disease grading (Grade II *vs*. Grade III-IV) did not show a significant difference between the two groups (p=0.266, Chi-Square test, **Table 2**). The cumulative incidence of grade II-IV aGVHD and grade III-IV aGVHD showed no significant difference between the two groups (p=0.089, p=0.228, Gray’s test, **Figures 1A, B**, respectively).

**Table 2:** Transplant outcomes.

Variables		ATG-T (n=46)	ATG-F (n=52)	p-value
**Median follow-up months (range)**		7.26 (2.6-24)	14.58 (4-24)	0.276
Infectious complications				
	Bacteremia	20 (43.5%)	24 (46.2%)	0.790
	CMV reactivation	36 (78.3%)	39 (75.0%)	0.704
	CMV disease	0	3 (5.8%)	0.098
	EBV reactivation	10 (21.7%)	4 (7.7%)	0.047
Other complications				
	VOD	8 (17.4%)	12 (23.1%)	0.486
	HC	11 (23.9%)	8 (15.4%)	0.287
**Hospitalization days (range)**		32.5 (21-256)	32.5 (14-248)	0.820
**Mortality Incidence (%)**		23 (50%)	25 (48.1%)	0.849
Death Cause				
	Relapse	9 (19.5%)	9 (17.3%)	0.952
	Infection	8 (17.3%)	8 (15.3%)	
	GVHD	5 (10.9%)	6 (11.53%)	
	Other	1 (2.2%)	2 (3.8%)	

aGVHD, Acute Graft Versus Host Disease; cGVHD, Chronic Graft Versus Host Disease; ANC, Absolute Neutrophil Count; PLT, Platelets; CMV, Cytomegalovirus; EBV, Epstein-Barr Virus; VOD, Veno-occlusive Disease; HC, Hemorrhagic Cystitis; DFS, Disease-Free Survival; GVHD, Graft Versus Host Disease.

Univariate analysis of the entire cohort did not show a statistically significant effect of type of ATG, GVHD prophylaxis (excluding ATG), type of donor, conditioning intensity, and patient’s age on the risk of aGVHD. In contrast, only HLA mismatching was associated with an increased risk for aGVHD (p=0.009, Log Rank test). Multivariate analysis (using Cox Regression model), incorporating HLA mismatching, ATG type and GVHD prophylaxis, revealed that donor HLA mismatching maintained its statistically significant effect on the risk for aGVHD (HR=2.118, 95% CI [1.119-4.010], p=0.021) while ATG type and GVHD prophylaxis were not statistically significant (Hazard ratios for all outcomes, incorporating ATG type into the Cox regression model, are summarized in **Table 3**).

**Table 3:** Hazard ratios of ATG type for different outcomes.

Outcome	HR	95% CI	P Value
Overall Survival	0.808	0.455-1.433	0.466
Relapse	0.579	0.258-1.299	0.185
NRM	0.912	0.452-1.842	0.798
AGVHD	0.729	0.398-1.338	0.308
CGVHD	1.137	0.500-2.585	0.760
GRFS	0.887	0.458-1.719	0.723

HR is given for ATG-F with ATG-T being the comparator.

Variables included in the model, determined by their significance in the univariate analysis (along with ATG type for each outcome), are as follows: OS, rDRI; HLA matching. Relapse, rDRI. NRM, HLA matching; demographics. AGVHD, HLA matching; GVHD prophylaxis type. CGVHD, Age, conditioning regimen. GRFS, rDRI, demographics.

NRM, Non-Relapse Mortality; aGVHD, Acute Graft Versus Host Disease; cGVHD, Chronic Graft Versus Host Disease; GRFS, cGVHD-free, relapse free survival.

cGVHD occurred in 10 (21.7%) and 15 (28.8%) patients in the ATG-T *vs*. ATG-F group, respectively (p=0.49, Fisher’s Exact test). Moderate-severe disease occurred in 10 (21.7%) *vs*. 13 (25%) patients, respectively (p=0.25, Fisher’s Exact test). No differences were found between the groups in the cumulative incidence for cGVHD (**Figure 1C**) and moderate-severe cGVHD (**Figure 1D**) (p=0.74 and p=0.965, respectively, Gray’s test). Univariate analysis revealed that myeloablative conditioning regimen and younger age were associated with a significant increased risk for cGVHD, while a history of aGVHD was associated with a borderline increased risk (p=0.068, Log Rank test). Donor-recipient gender mismatch and transplant source did not significantly affect the risk of developing cGVHD. In multivariate analysis, using the Cox Regression model, incorporating the significant factors identified in the univariate analysis (conditioning regimen and age), only myeloablative conditioning regimen was associated with an increased risk for cGVHD (results compared to MA regimen – RIC: HR=0.217, 95%CI [0.064-0.74], p=0.015, NMA: HR=0.113, 95%CI [0.015-0.841], p=0.033).

### Engraftment

**Figure 2:**
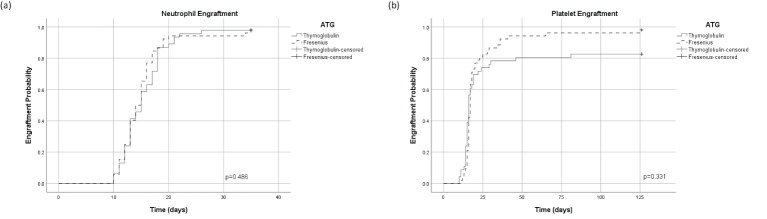
Cumulative incidence of engraftment. **(A)** Time dependent neutrophil engraftment. **(B)** Time dependent platelet engraftment.

There was no significant difference between the cohorts in the median time to neutrophil and platelet engraftment (**Figures 2A, B**, respectively). Forty-five patients (98%) and 51 patients (98%) in the ATG-T group *vs*. ATG-F group, have achieved neutrophil engraftment with a median time of 15 *vs*. 14 days, respectively (p=0.913). Thirty-Eight patients (82.6%) *vs*. 51 patients (98%) in the ATG-T group *vs*. ATG-F group have achieved platelet engraftment with a median time of 16 *vs*.17 days, respectively (p=0.360).

### Infections and other transplant related complications

No significant differences were observed in the incidence of bacteremia and CMV reactivation (**Table 2**). Bacteremia occurred in 43.5% *vs*. 46.2% (p=0.79), and CMV reactivation occurred in 78.3% *vs*. 75% (p=0.7, Chi-Square test) of patients in the ATG-T *vs*. ATG-F group, respectively. CMV disease, defined by the presence of clinical symptoms and/or signs together with documentation of CMV in tissue from the relevant organ [13], has occurred in three patients (5.8%) in the ATG-F group (CMV colitis and pneumonitis), with no documented cases in the ATG-T group (p=0.245, Fisher’s Exact test). EBV DNA viremia (detected by PCR) was observed in 21.7% of patients in the ATG-T group and 7.7% in the ATG-F group (p=0.047, Chi-Square test), with a single case of Post-Transplant Lymphoproliferative Disorder (PTLD) in the ATG-T group, associated with EBV-DNA viremia.

No significant differences were observed in other transplant related complications including incidence of veno-occlusive disease (VOD) or hemorrhagic cystitis (**Table 2**).

### Survival and relapse

**Figure 3:**
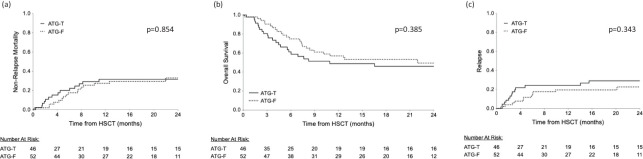
Cumulative survival rates. Similar survival rates are shown between the ATG-T and ATG-F groups. **(A)** relapse free survival and **(B)** overall survival and **(C)** relapse comparisons between the groups.

Median follow up time of the surviving patients was 10.91 months (range 2.6-24 months). OS was not significantly affected by ATG type, gender matching, transplant source (peripheral stem cells versus bone marrow), disease status at entry to transplant and aGVHD occurrence. However, HLA mismatching and a higher rDRI had a statistically significant negative effect on OS (p=0.008, p=0.017, respectively, Log Rank test). The presence of cGVHD was correlated with a significant better OS (p<0.001, Log rank test) and with a significantly lower incidence of relapse (p=0.008, Fisher’s Exact test). Using the Cox Regression model, incorporating ATG type as well as the significant pre transplant risk factors identified in the univariate analysis, donor HLA mismatching and rDRI were both associated with a significant hazard ratio for mortality (HR=1.997, 95% CI [1.120-3.562], p=0.019 and HR=1.899, 95% CI [1.070-3.372], p=0.028, respectively). Median follow up was 24 months in the ATG-T group *vs*. 21.5 months in the ATG-F group (p=0.485). At the end of follow-up, 23 patients (50%) in the ATG-T group were alive *vs*. 27 patients (51.9%) in the ATG-F group (p=0.849). The most common cause of death in both groups was relapse, with no significant difference in the cumulative incidence of NRM (**Figure 3A**, p=0.854). The distribution of causes of death also did not differ between the groups (**Table 2**). Furthermore, there was no significant difference in the cumulative incidence of OS and relapse between the two groups (p=0.385, **Figure 3B**; p=0.343, **Figure 3C**; respectively, Log Rank and Gray’s test).

**Figure 4:**
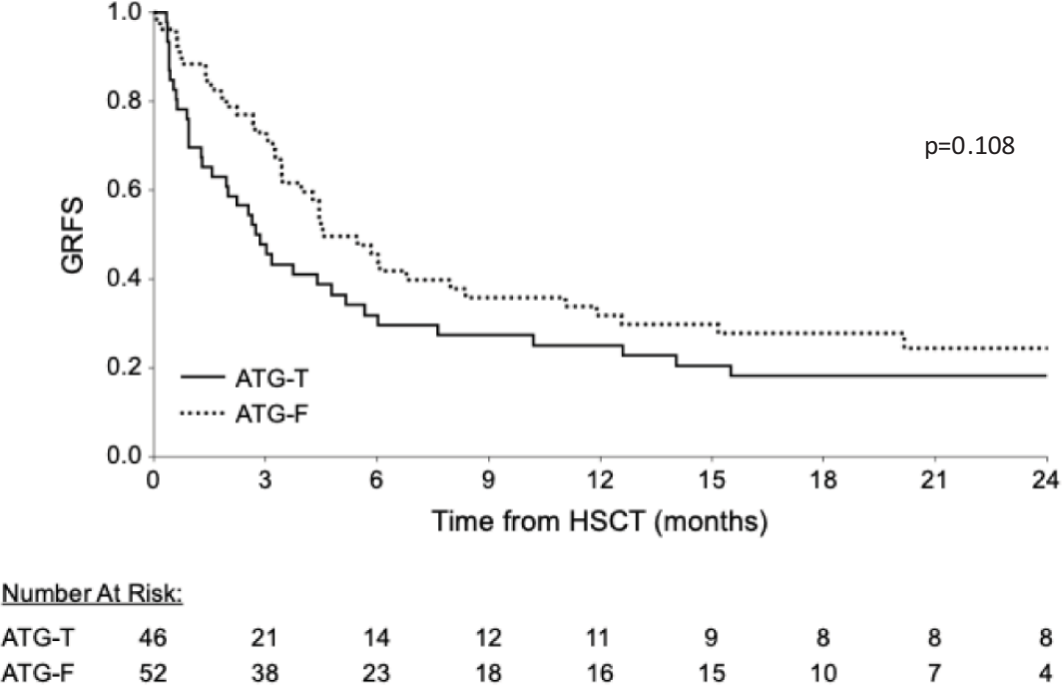
Cumulative GVHD Relapse Free Survival (GRFS). Similar cumulative GRFS are shown between the groups.

No difference was found between the groups regarding overall GRFS and moderate severe cGVHD-free, Relapse free survival (p=0.108, **Figure 4**, p=0.919, respectively; Log Rank test).

## Discussion

ATG-F has gained significant importance as GVHD prophylactic agent in patients undergoing HSCT in many centers. However, data regarding its optimal dose, as well as its efficacy and safety profile compared to ATG-T, is lacking. We present here a retrospective comparison of safety and efficacy outcomes between patients who were treated with ATG-T 10mg/kg and those who received ATG-F 15mg/kg at our medical center. We have found no significant difference in engraftment rates, cumulative risk for grade II-IV aGVHD and moderate-severe cGVHD, as well as DFS and OS. The two groups were highly comparable in demographic and baseline characteristics. However, there was a borderline significant higher incidence of HLA mismatch in the ATG-T group compared to the ATG-F group (p=0.052) and a significantly higher number of patients received single-agent cyclosporine in the ATG-T group (p<0.01). Notably, there was no difference in the rate of grade II-IV and III-IV aGVHD. Similarly to our results, other studies (as summarized in **Table 4**) comparing ATG-T and ATG-F at various dosing regimens did not report a disparity in GVHD incidence, either acute or chronic between the two agents (18, 23–25).

**Table 4:** Summary of published data regarding ATG-T and ATG-F.

First author name	Type of paper (retrospective vs prospective; single Vs. multicenter: phase 1,2, or 3: randomized vs. nonrandomized)	PBSC vs. BM	Donor type (MSD vs. MUD vs. Haplo vs. cord blood)	ATG Thymo dosage and no. of patients	ATG Fresenius dosage And no. of patients	Non-relapse mortality results	Infections	Acute GVHD incidence	Ch GVHD incidence	Relapse Incidence	Event free surviva	Overall survival
Wang L 2023 (25)	Single center retrospective	PBSC	MUD and MMUD	10 mg/kg n=107	20 mg/kg N=79	Similar rates of NRM	Higher rate of CMV viremia in ATG-T group 64.6% vs. 29.9%, p<0.001	Similar rates of aGVHD	Lower incidence of extensive cGVHD with ATG-T (p=0.01, HR=0.41)	Similar cumulative incidence of relapse.	Similar recurrence-free survival	Similar OS
Zhou L. 2020 (28)	Single center retrospective	Mixed	Haplo	7.5 MG/KG N=81	20 MG/KG N=35	Similar rates of TRM	Similar incidence of EBV infections	Similar rates of aGVHD	The cumulative incidence of any grade and limited cGVHD was higher in the ATG-T group (66% vs. 56% p=0.002 and 61.4 vs. 53.5%, p=0.007, respectively)	Similar cumulative incidence of relapse mortality	-	Similar OS (p=0.421)
Polverelli N. 2018 (19)	Single center retrospective	Both	MUD	7.5 MG/KG N=31	30 MG/KG N=46	Similar cumulative incidence of TRM	Similar infection rates	Similar rates of aGVHD	Similar rates of overall cGVHD but higher incidence of moderate- severe cVVHD in ATG-T (23% vs 8% p=0.03)	Similar RI	Similar DFS	Similar OS (p=0.58)
Huang W. 2016 (18)	Single center retrospective	PBSC	MUD	10 MG/KG N=56	20 MG/KG N=54	No significant differences between the groups in the 100-day or 3- year TRM rate	Similar infection rates	similar rates of aGVHD	lower rate of cGVHD in the ATG-F group (15% VS 33% respectively: *p* = 0.04)	Non-significant lower relapse rates in the ATG-F group and 5 years follow-up (20% vs 35% p=0.08 and 20% vs 40%; p=0.07, respectively)	ATG-T:3-y and 5-y DFS were 48% and 45% ATG-F- 67% and 67% *p* = 0.07 and *p* = 0.06	Similar 3y and 5 OS 58 vs 68%
Huang W. 2015 (26)	Single center retrospective	PBSC	MMUD	10 mg/kg N=23	20 mg/kg N=28	Similar NRM	Similar infection rates	Similar rates of aGVHD	Similar rates of cGVHD	Similar RI	Non-significant higher DFS rate in the ATG-F group, (45.7% vs 61.3%, p=0.08)	3-year OS rate was similar.
Paiano S. 2015 (24)	Single center retrospective	Both	Related, MUD, MMUD	7.5 MG/KG N=15	20 MG/KG N=15	Similar rates of TRM	Similar infection rates	Similar rates of aGVHD	Similar rates of cGVHD	Similar cumulative relapse incidence	Similar DFS	Similar OS
Basara N 2005 (23)	Multicenter retrospective	Both	MUD and MMUD	15 mg/kg (n=3), 10 mg/kg (n=28), 7.5 mg/kg (n=6), mg/kg (n=12)	45 mg/kg (n=11), 60 mg/kg (n=27)	Similar rates of TRM	-	Similar rates of aGVHD	The use of ATG-F was associated with lower incidence of cGVHD (p=0.05) which was not confirmed in multivariate analysis.		Projected 3-year LFS was higher in the ATG-F group (38% vs 21%, p=0.003)	OS was not

MUD, matched unrelated donor; MSD, matched sibling donor; NRM, non-relapse mortality; TRM, treatment-related mortality; RI, relapse incidience; GVHD, graft vs host disease.

There are conflicting reports on the efficacy of ATG-T and ATG-F in cGVHD prophylaxis. The rate of moderate-severe cGVHD with low dose ATG-F in our study was similar to previous reports (16, 17). Similar to the study by Huang et al. (26), we found no difference in cGVHD between the groups. Others have reported a lower incidence of cGVHD (18, 27) and moderate-severe cGVHD (19) in the ATG-F group. The higher dosage of ATG-F (20-30mg/kg), usage of quadruple GVHD prophylaxis and a selected homogeneous donor type (MUD or haploidentical donors) in these studies may be the cause for this discrepancy.

Survival analysis showed no significant differences in the cumulative incidence of OS or relapse between the two ATG prophylactic groups (p=0.385, p=0.343). Our findings align with previous studies conducted by Huang et al., Polverelli et al. and Zhou et al. showing a similar OS in patients undergoing HSCT from MUD (in the two first studies) and Haploidentical donors (in the latter study) treated with ATG-T versus ATG-F at various doses (10mg/kg, 7.5mg/kg, 7.5mg/kg and ATG-F 20mg/kg, 30mg/kg, 20 mg/kg, respectively) (18, 19, 28). In accordance with previously reported cohorts (20, 26, 27), we have observed that HLA mismatching and a higher rDRI are associated with lower OS in the entire cohort. In addition, cGVHD was associated with a favorable effect on OS and with a reduced incidence of relapse, consistent with previous literature linking between the graft-versus-leukemia (GVL) effect and cGVHD (27, 29).

We did not observe any significant difference in NRM, in accordance with previous studies. GRFS and moderate-severe chronic GRFS did not differ, contrary to Polverelli et al. (19), who found a statistically significant advantage to ATG-F administered in higher doses, in moderate-severe cGVHD-relapse-free survival, (p=0.042). In our study, safety analysis signals were limited to a higher incidence of EBV DNA viremia in the ATG-T group. Use of ATG is a known risk factor for EBV viremia and PTLD (30, 31). However, similar to previous reports, we have observed an exceptionally low incidence of PTLD (1%), with only one patient in the ATG-T group developing PTLD. Studies have indicated a dose-dependent risk, with reported viremia rate of 31% and EBV-associated disease rate of 29% in ATG-T dose of 7-8mg/kg and up to 50% viremia in doses above 10mg/kg (32). Similar to our findings, others have also reported a trend towards a higher rate of EBV viremia with ATG-T (7.5mg/kg) compared with ATG-F (20mg/kg) (28). No significant differences in other infectious complications were found between the groups, including CMV reactivation and disease, and bacteremia, consistent with other studies comparing ATG-T and ATG-F (18, 19, 28).

This study is limited by its retrospective nature, a relatively small cohort size, variability in hematological underlying disorders, and the comparison between different time periods. The comparison of different ATG types inherently involves distinct time periods, during which transplant practices, supportive care measures, and outcomes may have evolved. While the follow-up period was standardized to ensure comparability, we acknowledge that changes over time in transplant protocols and patient care could have influenced outcomes.

The ATG dosing strategies in our study were based on institutional practices during the respective time periods, reflecting evolving evidence and clinical safety concerns. While lower-dose ATG-F (15 mg/kg) was chosen to mitigate the risk of infectious complications and relapse, it remains below the doses traditionally used in earlier studies. Furthermore, ATG dosing was not based on pharmacokinetics or absolute lymphocyte counts, as suggested by recent studies.

However, to the best of our knowledge this is the first report comparing low dose ATG-F (15mg/kg) with ATG-T at a dose of 10mg/kg. Moreover, there is a relatively high incidence of grade III-IV acute GVHD in both study groups. This can be attributed to the lower utilization of methotrexate (MTX) in our standard GVHD prophylaxis protocol during the documented years. Furthermore, donor lymphocyte exposure to ATG plays a pivotal role in GVHD risk. Unfortunately, our study did not encompass pharmacokinetic measurements, preventing us from investigating this critical factor thoroughly. Admiraal and colleagues’ study suggested that customizing ATG dosing based on absolute lymphocyte counts may yield superior target achievement when compared to weight-based dosing (33). These limitations pose challenges on the generalizability of our findings.

Despite these limitations, the lack of adverse signals in our study is encouraging and suggests that the use of low dose ATG-F for GVHD prophylaxis, at a dose of 15mg/kg, is safe. Nonetheless, to draw definitive conclusions and establish the optimal type and dose of ATG for GVHD prophylaxis, a randomized controlled prospective study is needed. Such a study should incorporate MTX in MA GVHD prophylaxis protocols and include comprehensive pharmacokinetic assessments of ATG. This would allow for precise evaluation of the relationship between ATG exposure, absolute lymphocyte counts, and clinical outcomes such as GVHD incidence, relapse rate, and overall survival. Furthermore, post-transplant Cyclophosphamide (PT-Cy) has emerged as a promising agent for GVHD-prophylaxis. Retrospective studies have compared ATG to PT-Cy (34, 35) showing conflicting results. Strategies combining ATG and PT-Cy have been the subject of recent investigation (36). In haploidentical or unrelated donor settings, the addition of reduced doses of PT-Cy to ATG has shown promise. These findings suggest that the combination of ATG and PT-Cy can be a valuable strategy emphasizing the need to define the dosage and type of administered ATG.

In summary, while this study provides valuable insights into the safety and efficacy of low-dose ATG-F compared to ATG-T, further research is needed to validate these findings and guide clinical decision-making effectively. Prospective studies with larger patient cohorts and controlled designs will help to better understand the potential benefits and risks of different ATG dosing regimens for GVHD prophylaxis in hematopoietic stem cell transplantation.

## Data Availability

The original contributions presented in the study are included in the article/supplementary material. Further inquiries can be directed to the corresponding authors.
